# Remimazolam provides better hemodynamic stability than propofol in hypertensive surgical patients: a randomized single-blinded trial

**DOI:** 10.1007/s00540-025-03644-1

**Published:** 2025-12-17

**Authors:** Ping Wang, Dongying Chen, Haiyan Yu, Yanyan Sun, Jun Xiong

**Affiliations:** Department of Anesthesiology, Shenzhen University General Hospital, Shenzhen University, Shenzhen, 518055 Guangdong Province China

**Keywords:** Hypertension, Hemodynamics, Propofol, Benzodiazepines, Intraoperative complications, Anesthesia, Intravenous

## Abstract

**Purpose:**

Hypertensive surgical patients face heightened perioperative cardiovascular risk, and propofol often induces hypotension. This trial aimed to compare remimazolam and propofol for hemodynamic stability in hypertensive patients undergoing non-cardiac surgery, and explore underlying cardiac and vascular mechanisms via continuous monitoring.

**Methods:**

This randomized single-blind trial enrolled 122 adults with controlled hypertension undergoing elective non-cardiac surgery, assigned to remimazolam-based or propofol-based total intravenous anesthesia. Primary outcomes were intraoperative hypotension episodes (mean arterial pressure < 65 mmHg or a > 20% decrease from baseline), norepinephrine bolus frequency and total dose. Hemodynamics were continuously tracked by Continuous Non-Invasive Arterial Pressure Monitor 500, with anesthesia depth maintained at a bispectral index of 40–60.

**Results:**

Baseline characteristics were comparable. The remimazolam group had fewer hypotension episodes (remimazolam group vs. propofol group, median episodes [interquartile range, IQR], 2 [0–3] vs. 3 [1–5]; *p* = 0.003), fewer norepinephrine boluses (1 [0–3] vs. 3 [1–5]; *p* = 0.001), and lower total dose (8 µg [0–24] vs. 24 µg [8–40]; *p* < 0.001). Remimazolam showed non-significant trends toward smaller reductions in cardiac output (CO, − 8.10% vs. − 13.10%, *p* = 0.35) and systemic vascular resistance (SVR, − 10.83% vs. − 14.91%, *p* = 0.46). Extubation time and post-anesthesia care unit stay were similar.

**Conclusion:**

For hypertensive patients, remimazolam provides superior hemodynamic stability over propofol, evidenced by fewer hypotensive episodes, reduced norepinephrine requirements, and attenuated perturbations in CO and SVR, without delaying recovery. It represents a valuable anesthetic alternative for this high-risk cohort.

## Introduction

Hypertension is highly prevalent among surgery patients and significantly increases perioperative cardiovascular risk [1–3]. This population is particularly vulnerable to intraoperative hypotension, an event strongly and independently associated with adverse postoperative outcomes, including myocardial and kidney injury [4–6]. Therefore, maintaining hemodynamic stability is a critical goal in their anesthetic management.

Propofol, the current gold standard for intravenous anesthesia, is well-known for its dose-dependent cardiovascular depression, frequently causing hypotension [7, 8]. This pharmacologic drawback drives the search for safer alternatives with a more favorable hemodynamic profile.

Remimazolam, a novel ultrashort-acting benzodiazepine, has emerged as a promising agent. It combines rapid onset and offset with a theoretically neutral hemodynamic effect [9]. Preliminary clinical studies, primarily in procedural sedation, have demonstrated a significantly lower incidence of hypotension compared to propofol [7, 10]. Evidence also supports its efficacy and stability for general anesthesia induction and maintenance [11–13].

However, few studies have compared the hemodynamic effects of remimazolam versus propofol specifically in hypertensive surgical patients, a population with heightened cardiovascular vulnerability and a lower threshold for instability. Moreover, it remains unclear whether any observed hemodynamic differences between these agents stem from variations in their impact on cardiac function, systemic vascular resistance, or a combination of both. No randomized controlled trial has comprehensively evaluated remimazolam against propofol for total intravenous anesthesia (TIVA) in this high-risk cohort using detailed, continuous hemodynamic monitoring capable of dissecting these mechanisms.

We therefore conducted this randomized controlled trial to determine whether remimazolam provides superior hemodynamic stability compared to propofol in hypertensive adults undergoing non-cardiac surgery. To precisely capture dynamic hemodynamic changes often missed by conventional intermittent measurements and to investigate the potential cardiac and vascular mechanisms underlying any differences, we employed high-fidelity continuous hemodynamic monitoring using the Continuous Non-Invasive Arterial Pressure (CNAP) Monitor 500 system (CNSystems, Graz, Austria). This approach addresses a significant gap in optimizing anesthesia management for this vulnerable population.

## Methods

### Ethics

This randomized, single-blind trial compared remimazolam versus propofol for TIVA in hypertensive adults between April and September 2025. Approved by the hospital’s Institutional Review Board (no. KYLLHS-20230205B-E) and registered at the Chinese Clinical Trial Registry (ChiCTR2500100144), the study adhered to the Declaration of Helsinki and ICH-GCP guidelines. All participants provided preoperative written consent.

### Study population

Inclusion criteria: Adult patients (18–65 years) with a history of hypertension and American Society of Anesthesiologists (ASA) physical status II-III, undergoing elective procedures (general, urological, or gynecological) under general anesthesia (anticipated duration ≤ 3 h, estimated blood loss < 200 mL).

Exclusion criteria: ASA physical status ≥ IV (with severe cardiovascular, respiratory, or endocrine system diseases, or major organ failure); known allergy to study medications or chronic sedative use prior to enrollment; history of psychiatric disorders or cognitive impairment; pregnancy or planned pregnancy; preoperative airway comorbidities (e.g., asthma, sleep apnea); myasthenia gravis; or chronic renal/hepatic dysfunction.

### Randomization and blinding

Patients were randomized 1:1 to receive either remimazolam or propofol. Randomization was performed using a computer-generated random number prior to induction. Due to the distinct appearance and administration protocols of the two investigational drugs, blinding of the attending anesthesiologists was not feasible. However, all personnel responsible for outcome data collection and statistical analysis remained blinded to group allocation throughout the study.

### Study protocol

Standard intraoperative monitoring comprised electrocardiography, pulse oximetry (SpO_2_), and continuous hemodynamic assessment via CNAP recording systolic blood pressure (SBP), diastolic blood pressure (DBP), mean arterial pressure (MAP), heart rate (HR), cardiac output (CO), systemic vascular resistance (SVR), pulse pressure variation (PPV), and stroke volume (SV). Depth of anesthesia was objectively monitored using bispectral index (BIS; Covidien, Mansfield, MA, USA) with a target range of 40–60 [14].

Patients were randomly assigned to receive either remimazolam- or propofol-based TIVA. In the remimazolam group (Group R), induction was initiated with a continuous intravenous infusion at 6 mg/kg/h [15] for up to 3 min. If loss of consciousness (LOC), defined as BIS ≤ 60, was not achieved within this period, supplemental doses of 12 mg/kg/h could be administered over ≤ 1 min until LOC was attained. Anesthesia was maintained with remimazolam 0.5–3.0 mg/kg/h infusion. In the propofol group (Group P), induction was accomplished with a bolus dose of 2–2.5 mg/kg [15] until LOC (same criteria), followed by continuous infusion at 3–6 mg/kg/h. Both groups received standardized adjunct medications including sufentanil (0.2–0.4 µg/kg) and rocuronium (0.4–1.0 mg/kg) for induction, with remifentanil (0.05–0.15 µg/kg/min) for maintenance. Anesthesia depth was continuously monitored by BIS, with intubation performed when BIS ≤ 60 was achieved. Throughout the procedure, anesthetic doses were titrated to maintain BIS values between 40 and 60, while ensuring hemodynamic stability within ± 20% of MAP. While BIS maintenance between 40 and 60 was the target, transient deviations were anticipated and managed per clinical routine. Hypotension was defined as MAP < 65 mmHg or a > 20% decrease from baseline [16], whichever occurred first, and was immediately treated with norepinephrine boluses (8–16 µg) as needed [17]. The bolus was prepared by diluting 2 mg of norepinephrine in 250 ml of normal saline, yielding a concentration of 8 µg/ml. A bolus of 1–2 ml of this solution (8–16 µg, approximately equivalent to 0.12–0.24 µg/kg based on the average patient weight) was administered. This standardized rescue protocol ensured patient safety but may have normalized blood pressure at subsequent discrete measurement timepoints. In the remimazolam group, the use of flumazenil was permitted at the discretion of the attending anesthesiologist, based on clinical assessment of patient emergence and operating room workflow needs. This approach was designed to reflect real-world practice while prioritizing the observation of spontaneous recovery. No specific reversal agent was available for the propofol group.

### Data collection protocol

Demographic and baseline characteristics, including age, gender, body mass index (BMI), ASA physical status, and antihypertensive medication history (use and classes), were collected. Baseline hemodynamic parameters were defined as the average of three preoperative readings in the operating room and included the following: SBP, DBP, MAP, and HR. Intraoperative variables encompassed anesthesia duration, surgical time, total intravenous fluid administration, and estimated blood loss.

Hemodynamic parameters (SBP, DBP, MAP, HR, CO, SVR, PPV, and SV) and BIS values were documented at predefined timepoints: before induction (Baseline T0), at tracheal intubation (T1), initial surgical incision (T2), and subsequently at 30-min intervals until the 3-h mark (or procedure completion if earlier)(T3–T8), followed by final wound closure (T9) and extubation (T10).

Outcome measures included: intraoperative hypotension episodes (defined as MAP < 65 mmHg or a > 20% decrease from baseline), norepinephrine requirements (both bolus counts and total dosage), time from drug discontinuation to extubation, and post-anesthesia care unit (PACU) length of stay. All data were collected by trained anesthesiologists using standardized case report forms.

### Study endpoint

Primary outcomes:

1. Intraoperative hypotension episodes,
2. Frequency of norepinephrine administration, and
3. Total dosage of norepinephrine required.

Secondary outcomes:

1. Hemodynamic measurements at predefined serial timepoints,
2. Mean BIS values at the same timepoints,
3. Time to extubation (from drug discontinuation to tracheal tube removal), and
4. Length of stay in PACU.

### Sample size calculation

The sample size was calculated using PASS Statistics (15.0.5, Kaysville, Utah, USA), based on a negative binomial model. The calculation was powered to detect a difference in the mean number of hypotensive episodes, which was set at 0.75 for the propofol group and 0.23 for the remimazolam group based on a prior study [17]. The dispersion parameter (*k*) was set to 1.0, as estimated from our internal pilot study (*n* = 15 per group) [18]. With a two-sided alpha of 0.05, a power of 80%, and assuming a 15% dropout rate, the analysis indicated a minimum requirement of 56 patients per group (112 total). To account for a potentially higher dropout rate, we ultimately planned to enroll 61 patients per group, resulting in a total sample size of 122 patients.

### Statistical analysis

All statistical analyses were performed using SPSS Statistics (26.0; IBM Corp., Armonk, NY, USA). Continuous variables were presented as mean ± standard deviation (SD) or median [interquartile range, IQR] as appropriate. The normality of distribution for all continuous variables, including the number of hypotensive episodes and norepinephrine requirements, was formally assessed using the Shapiro–Wilk test. As these primary outcome variables were found to be non-normally distributed, between-group comparisons were performed using the non-parametric Mann–Whitney *U* test. Categorical variables were compared using the *χ*^2^ test or Fisher’s exact test. All statistical tests were two-tailed, with a *p* value < 0.05 considered significant.

In this study, 2 patients had missing data for some secondary outcome variables due to factors independent of the study intervention. Multiple imputation [19] was used to handle missing data, with the model incorporating baseline characteristics, surgical details, and all outcome measures. A complete case analysis was performed as a sensitivity analysis, and detailed results are available in the supplementary materials to confirm the robustness of our findings.

Data of secondary outcomes from time points T7 and T8 were excluded from the final statistical analysis due to the completion of surgeries in a majority of patients prior to these time points. This precluded meaningful group comparisons at T7 and T8.

## Results

### Patient recruitment and baseline characteristics

**Fig. 1:**
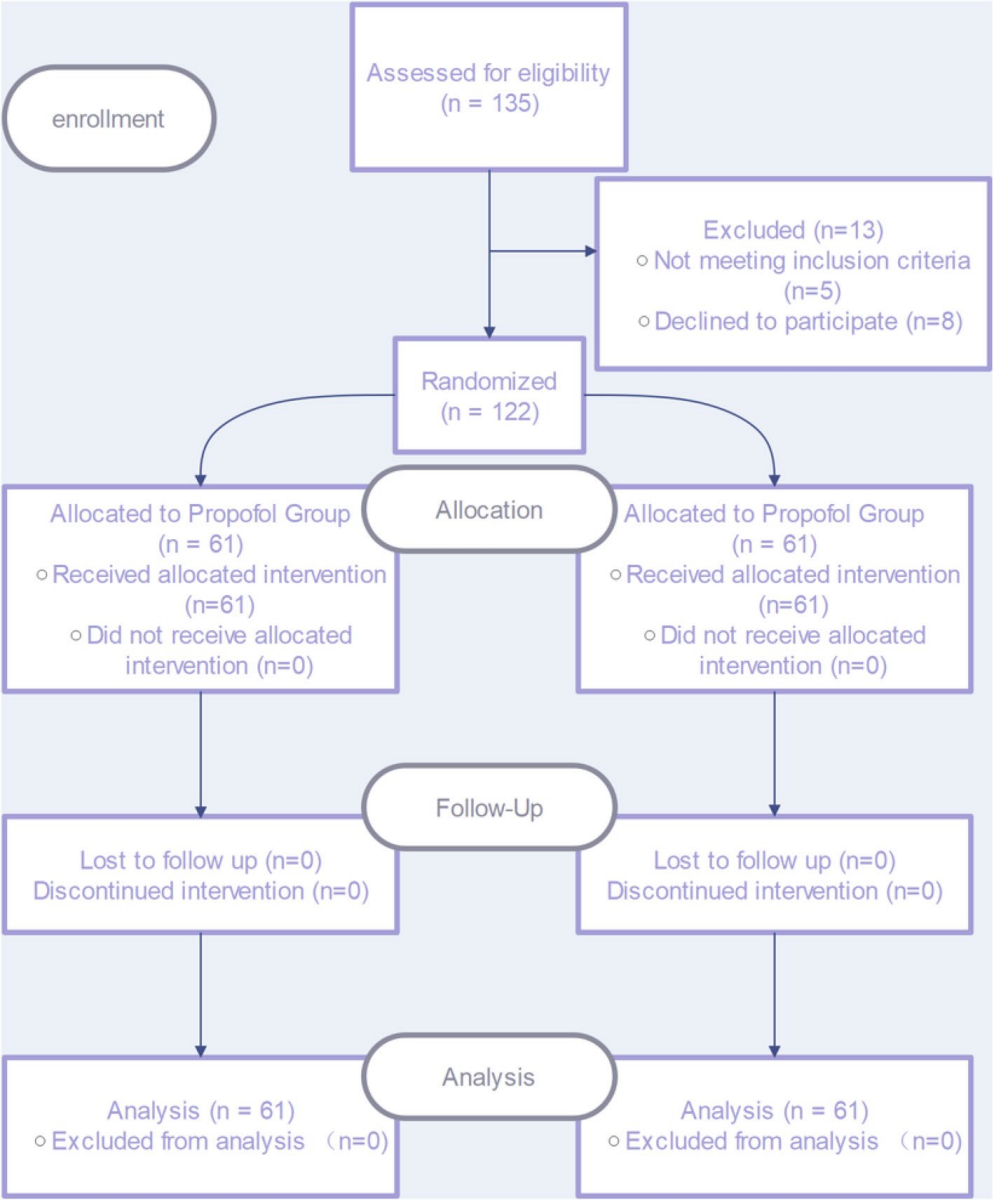
CONSORT flow diagram of the randomized controlled trial

A total of 135 patients were assessed for eligibility. After exclusions and randomization, 122 patients (61 per group) were included in the final analysis (Fig. 1).

The baseline demographic, clinical, and basic hemodynamic parameters were well-balanced between the remimazolam and propofol groups, with no statistically significant differences observed (Table 1). Intraoperative management indicators (e.g., anesthesia duration, fluid infusion volume) and adjunctive drug dosages (e.g., sufentanil, remifentanil) were also comparable between groups (Table 2).

**Table 1:** Comparison of baseline characteristics and antihypertensive medication

Variables	Group R (*n* = 61)	Group P (*n* = 61)	*p* values
Demographics			
Age (years)	57.30 ± 7.39	55.02 ± 7.51	0.10
Gender (M/F)	36 (59)/25 (41)	40 (66)/21 (34)	0.58
Height (cm)	166.21 ± 6.29	164.82 ± 6.94	0.25
Weight (kg)	65.86 ± 10.65	66.85 ± 10.04	0.60
BMI (kg/m^2^)	24.14 ± 2.80	24.13 ± 2.86	0.79
ASA (II/III)	52 (85)/9 (15)	54 (89)/7 (11)	0.79
Comorbidities			
Diabetes mellitus	16 (26.2)	10 (16.4)	0.27
Coronary heart disease	2 (3.3)	4 (6.6)	0.68
Cerebral infarction	6 (9.8)	2 (3.3)	0.36
Antihypertensive medication use			
No antihypertensive medication	5 (8.2)	5 (8.2)	1.00
Monotherapy	35 (57.4)	29 (47.5)	0.41
Combination therapy (≥ 2 drugs)	21 (34.4)	27 (44.3)	0.35
Classes of medications			
ARB	25 (41.0)	34 (55.7)	0.14
ACEI	4 (6.6)	5 (8.2)	1.00
CCB	38 (62.3)	36 (59.0)	0.78
Diuretics	0 (0.0)	1 (1.6)	1.00
β-blocker	14 (23.0)	12 (19.7)	0.77
Others	1 (1.7)	0 (0.0)	1.00
Basic hemodynamic parameters			
SBP (mmHg)	126.75 ± 18.44	128.37 ± 19.63	0.64
DBP (mmHg)	78.27 ± 11.60	78.83 ± 14.63	0.81
MAP (mmHg)	96.30 ± 14.53	97.98 ± 16.85	0.56
HR (bpm)	71.63 ± 9.25	68.90 ± 8.83	0.10

Data are presented as mean ± standard deviation, or number of patients (%)

*p* values were derived from independent *t* tests (continuous data) or Chi-square or Fisher’s exact tests (categorical data)

*BMI* body mass index; *ASA* American Society of Anaesthesiologists; *ARB* Angiotensin II receptor blocker; *ACEI* Angiotensin-converting enzyme inhibitor; *CCB* Calcium channel blocker; *SBP* Systolic Blood Pressure; *DBP* Diastolic Blood Pressure; *MAP* Mean Arterial Pressure; *HR* Heart Rate

**Table 2:** Comparison of intraoperative management and drug dosages

Variable	Group R (*n* = 61)	Group P (*n* = 61)	*p* values
Intraoperative management			
Anesthesia Duration (min)	109.10 ± 40.50	109.30 ± 40.15	0.58
Surgery Duration (min)	92.25 ± 37.13	95.28 ± 30.94	0.47
Fluid infusion volume (ml)	821.67 ± 353.39	873.97 ± 275.09	0.12
Estimated blood loss (ml)	2.80 ± 3.07	2.77 ± 2.23	0.61
Study drug dosages			
Induction dose (mg/kg)	0.33 ± 0.06	2.12 ± 0.31	N/A
Maintenance infusion rate (mg/kg/h)	1.17 ± 0.24	4.76 ± 0.67	N/A
Concomitant drug dosages			
Sufentanil (µg/kg)	0.23 ± 0.06	0.24 ± 0.07	0.82
Remifentanil (µg/kg/min)	0.068 ± 0.018	0.074 ± 0.026	0.35
Rocuronium (mg/kg)	0.47 ± 0.18	0.48 ± 0.28	0.46

Data are expressed as mean ± standard deviation

Continuous data were compared between groups using the independent *t* tests, with *p* < 0.05 considered statistically significant

### Primary outcomes: hypotension episodes and norepinephrine requirements

The remimazolam group demonstrated significantly superior hemodynamic stability across all primary outcomes. The intraoperative hypotension episodes were markedly lower in the remimazolam group compared to the propofol group (2 [0–3] vs. 3 [1–5]; *p* = 0.003). Consequently, both the frequency of norepinephrine bolus administration (1 [0–3] vs. 3 [1–5]; *p* = 0.001) and the total norepinephrine dose (8 μg [0–24] vs. 24 μg [8–40]; *p* < 0.001) were significantly reduced in the remimazolam group (Table 3).

**Table 3:** Comparison of primary outcomes

Outcome measures	Group R	Group P	Effect size (*r*)	*p* values
Hypotensive episodes (times)	2 [0–3]	3 [1–5]	− 0.31	0.003^*^
Norepinephrine boluses (times)	1 [0–3]	3 [1–5]	− 0.35	0.001^**^
Total norepinephrine dose (µg)	8 [0–24]	24 [8–40]	− 0.36	< 0.001^***^

Data are expressed as median, [IQR]

*p* values were calculated using the Mann–Whitney *U* test with *p* < 0.05 considered statistically significant. *p = 0.003, **p = 0.001, ***p < 0.001

The effect size was quantified by the correlation coefficient *r*, with |*r*|≥ 0.3 indicating medium size

*IQR* Interquartile Range

The results of the sensitivity analysis (complete case analysis) were consistent with the primary analysis, with the same trend of between-group differences observed and no substantial changes in statistical significance, indicating the robustness of the conclusions (detailed data see supplementary materials).

### Secondary outcomes

#### Time to extubation, length of PACU stay and depth of anesthesia (BIS value)

**Fig. 2:**
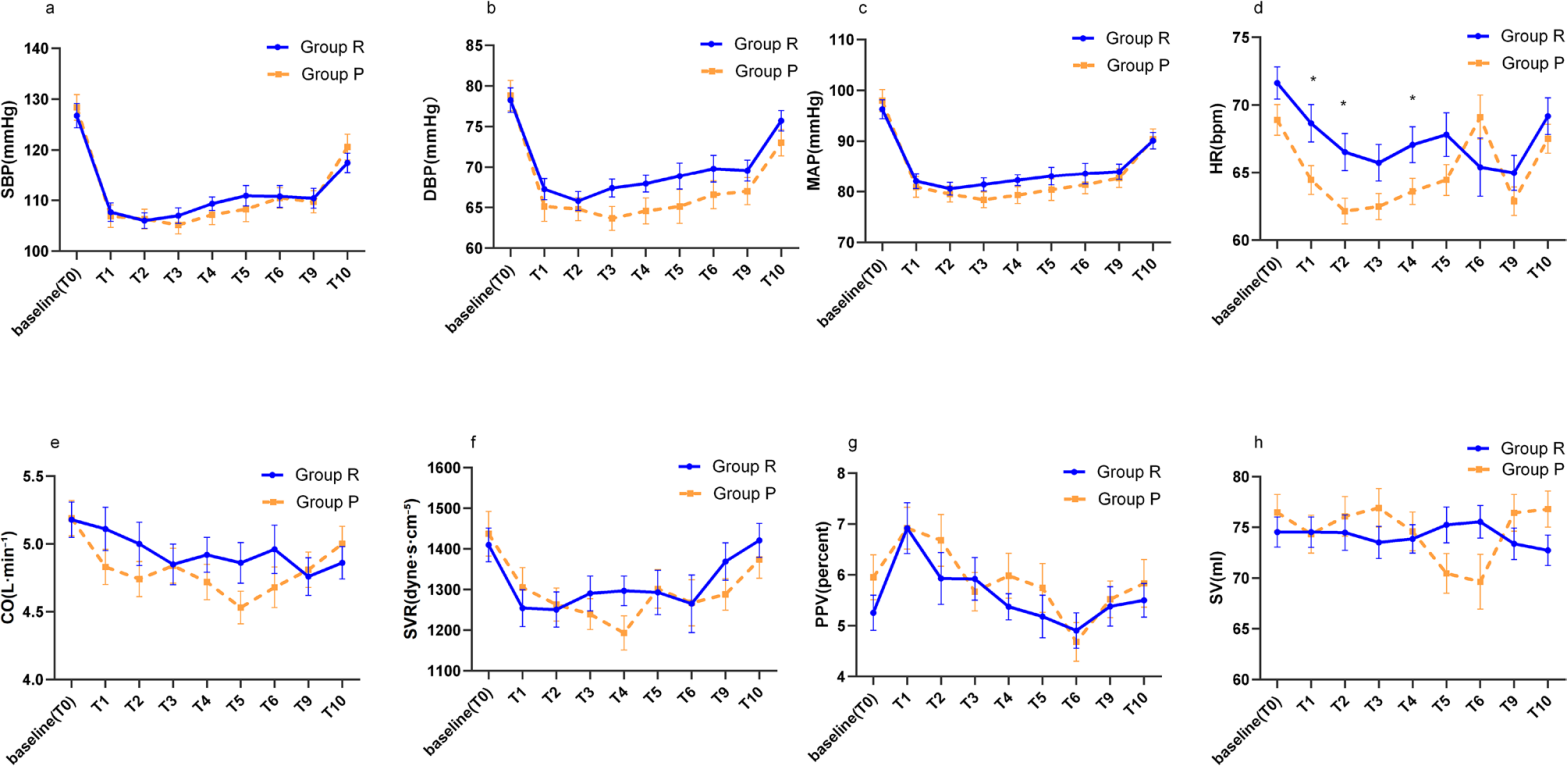
Intraoperative trends of hemodynamic parameters. The blue solid line represents the remimazolam group (Group R), and the orange dashed line represents the propofol group (Group P). **a** Systolic blood pressure (SBP), **b** Diastolic blood pressure (DBP), **c** Mean arterial pressure (MAP), **d** Heart rate (HR); Group P exhibited a significantly lower HR compared to Group R at time points T1 (intubation), T2 (initial surgical incision), and T4 (60 min post-incision) (*p < 0.05 for all comparisons), **e** Cardiac output (CO), **f** Systemic vascular resistance (SVR), **g** Stroke volume variation (SVV), and **h** Stroke volume (SV). No statistically significant differences were observed in the remaining parameters (SBP, DBP, MAP, CO, SVR, SVV, SV) at the predefined time points

There were no statistically significant differences between the two groups in the time from drug discontinuation to extubation or the length of PACU stay (Table 4). The depth of anesthesia, as monitored by the BIS, was effectively maintained within the target range (40–60) in both groups throughout the operation. Although statistically significant differences in BIS values were observed at specific time points (e.g., T1, T3), these transient discrepancies did not translate into a clinically meaningful impact on emergence outcomes (Table 4 and Fig. 2).

**Table 4:** Comparison of secondary outcomes

Secondary outcome measures	Group R (*n* = 61)	Group P (*n* = 61)	*p* values
Time to extubation (min)	12.52 ± 4.38	12.00 ± 4.96	0.46
length of PACU stay (min)	36.08 ± 8.64	37.84 ± 9.54	0.20
BIS			
T0	95.87 ± 5.32	96.11 ± 2.04	0.69
T1	51.57 ± 6.22	47.41 ± 6.02	< 0.001^***^
T2	51.59 ± 7.11	49.25 ± 7.07	0.07
T3	51.13 ± 6.46	48.57 ± 6.07	0.04^*^
T4	50.32 ± 6.80	47.53 ± 6.14	0.05
T5	50.77 ± 2.87	47.09 ± 5.04	0.01^*^
T6	50.12 ± 3.36	48.71 ± 3.40	0.21
T9	57.07 ± 6.43	55.41 ± 6.94	0.23
T10	78.19 ± 5.63	78.20 ± 6.90	0.95

Data are presented as mean ± standard deviation

*p* values were derived from independent t-tests with *p* < 0.05 considered statistically significant. *p < 0.05, ***p < 0.001

*PACU* Post-Anesthesia Care Unit; *BIS* Bispectral Index

#### Hemodynamic indicators

**Fig. 3:**
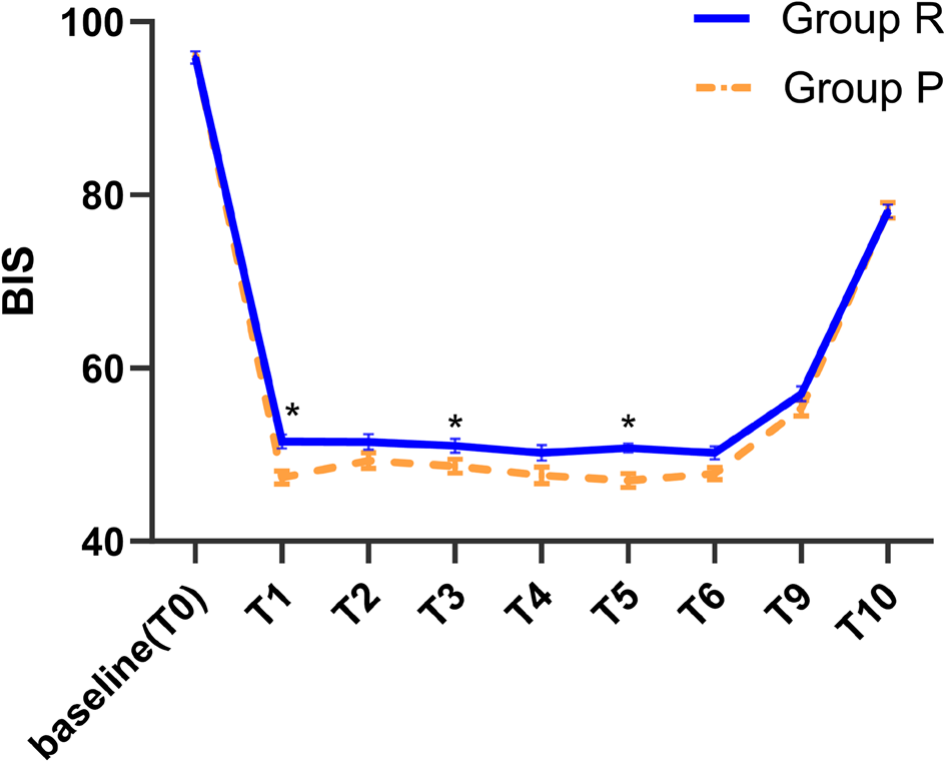
Intraoperative trends of bispectral index (BIS). Line definitions are consistent with Fig. 3. Group R maintained a significantly higher BIS compared to Group P at time points T1 (intubation), T3 (30 min post-incision), and T5 (90 min post-incision) (*p < 0.05 for all comparisons). Both groups exhibited BIS values within the target range (40–60) throughout the procedure, with a rapid increase at T10 (extubation)

Continuous hemodynamic monitoring revealed distinct patterns between the two anesthetic regimens. Trends for SBP, DBP, MAP, CO, SVR, PPV, and SV were largely comparable between groups throughout the procedure (Fig. 3). However, the propofol group exhibited a transient but statistically significant reduction in heart rate (HR) at specific time points (T1, T2, T4) compared to the remimazolam group (T1: *p* = 0.028; T2: *p* = 0.012; T4: *p* = 0.037). Furthermore, analysis of maximum change from baseline revealed that propofol was associated with more pronounced reductions in both CO and SVR following anesthesia induction, as well as SV (Table 5).

**Table 5:** Comparison of intraoperative CO and SVR between two groups

	Group	T0	T1	T4	T5	T9	*p* values	Intragroup Maximum Rate of Change (%) *
CO (L/min)	R	5.18 ± 1.03	5.11 ± 1.21	4.92 ± 0.98	4.85 ± 0.86	4.76 ± 1.03	0.35	− 8.10(T9 vs T0)
	P	5.19 ± 1.03	4.83 ± 1.03	4.76 ± 1.00	4.51 ± 0.70	4.81 ± 0.99		− 13.10(T5 vs T0)
SVR (dyn·s/cm^−5^)	R	1408.90 ± 318.40	1256.33 ± 348.32	1299.09 ± 278.35	1319.90 ± 361.84	1369.47 ± 356.08	0.46	− 10.83(T1 vs T0)
	P	1434.21 ± 424.52	1306.93 ± 379.92	1220.43 ± 326.85	1369.83 ± 320.09	1289.68 ± 297.07		− 14.91(T4 vs T0)
PPV (percent)	R	5.21 ± 2.46	6.46 ± 3.40	5.37 ± 2.02	5.17 ± 2.08	5.36 ± 3.02	0.26	23.99(T1 vs T0)
	P	5.93 ± 2.70	6.92 ± 3.16	5.98 ± 3.27	5.67 ± 2.46	5.48 ± 2.79		16.69(T1 vs T0)
SV (ml)	R	74.56 ± 11.56	74.43 ± 11.46	73.70 ± 10.71	74.46 ± 10.59	73.38 ± 11.95	0.39	− 1.58(T9 vs T0)
	P	76.44 ± 13.70	74.21 ± 14.42	74.49 ± 14.83	70.56 ± 11.50	76.38 ± 14.01		− 7.69(T5 vs T0)

Data are presented as mean ± standard deviation

^*^Intragroup maximum rate of change was calculated as (Minimum value of the index at any time point—Baseline value at T0)/Baseline value at T0 × 100%, with negative values representing a decrease

*p* values were intergroup comparison derived from Repeated-Measures ANOVA tests with *p* < 0.05 considered statistically significant

*CO* cardiac output; *SVR* Systemic Vascular Resistance; *PPV* Pulse Pressure Variation; *SV* Stroke Volume

## Discussion

This randomized controlled trial demonstrates that remimazolam provides superior hemodynamic stability compared to propofol in hypertensive patients undergoing non-cardiac surgery, as evidenced by significantly fewer hypotension episodes and a marked reduction in norepinephrine requirements.

Although previous studies have compared the hemodynamic effects of remimazolam with propfol [8, 20–22], most of these studies rely on discrete blood pressure measurements, which makes it difficult to capture the dynamic process of intraoperative hemodynamic fluctuations and, more importantly, prevents further elucidation of CO and SVR mechanisms underlying blood pressure changes.

Emerging evidence has demonstrated that high-fidelity continuous hemodynamic monitoring enables more accurate capture of dynamic hemodynamic changes and better elucidation of underlying regulatory mechanisms compared to discrete measurements [23, 24]. The principal novelty of the present study lies in the adoption of CNAP Monitor 500, which enables real-time and continuous collection of intraoperative hemodynamic parameters. This approach not only allows dynamic tracking of blood pressure changes but also further decomposes the contributions of core indicators such as CO and SVR, thereby suggesting in hypertensive patients that the hemodynamic stability maintained by remimazolam is likely attributed to its smaller impact on CO and SVR, coupled with a more favorable HR compensatory response. To substantiate this speculative mechanism, we further analyzed the dynamic changes of core hemodynamic parameters.

Our analysis revealed that during the anesthetic maintenance phase, the maximum reductions in CO and SVR were numerically smaller in the remimazolam group. Although this difference did not achieve statistical significance, the consistent directional trend across these two key hemodynamic parameters suggests a less profound initial hemodynamic disturbance. Notably, this attenuated hemodynamic insult was accompanied by a significantly higher HR in the remimazolam group at multiple time points. Collectively, these observations point to a plausible mechanism underlying remimazolam’s advantage: it induces milder hemodynamic perturbation while preserving physiological compensatory responses, thereby supporting greater overall stability.

This advantage of remimazolam becomes more distinct when contrasted with propofol, which was well-documented to cause hemodynamic instability in hypertensive patients via synergistic direct myocardial depression and systemic vasodilation [25–27]. In our study, CNAP monitoring quantified this insult: the propofol group exhibited more pronounced maximum reductions in CO and SVR during the anesthetic maintenance phase. Mechanistically, remimazolam’s superiority lies in its attenuated impact on these two key blood pressure determinants: it not only resulted in a numerically smaller maximum CO reduction (partly attributable to better maintained preload, suggested by less SV fluctuation via muted effects on venous capacitance) but also might have caused a less pronounced SVR decrease compared to propofol.

Notably, our observation of a trend toward better SV preservation with remimazolam presents a nuanced contrast to the findings of Sekiguchi et al. [28], who reported a numerical trend in the opposite direction. It is crucial to emphasize that both studies are aligned on the core finding: no statistically significant difference in SV or CO was established between the anesthetic agents. The divergence thus lies solely in the direction of non-significant numerical trends. This discrepancy in trends may be attributable to methodological differences, particularly the anesthetic regimen. The study by Sekiguchi et al. [28] employed propofol target-controlled infusion (TCI) combined with a high-dose remifentanil infusion. The known synergistic effect of high-dose remifentanil in reducing cardiac preload and contractility [29] may have been more pronounced in their remimazolam group, unmasking a greater SV reduction trend. In contrast, our protocol, which utilized a standard propofol continuous infusion alongside a lower dose of remifentanil, likely resulted in a weaker synergistic depressive effect, thereby allowing remimazolam’s inherently milder impact on preload to manifest as a more favorable SV trend.

Beyond the favorable SV trend, our data highlight a second potential mechanism underlying remimazolam’s hemodynamic advantage: compensatory HR elevation. The significantly higher HR in the remimazolam group at intubation and incision may reflect a preserved baroreceptor-mediated tachycardic response. In contrast, propofol is known to suppress the baroreflex, blunting this essential compensatory mechanism [30]. Our findings align with those of Tsukimoto et al. [31], who used HR variability analysis to demonstrate that remimazolam, unlike propofol, does not alter the balance of the autonomic nervous system, thereby permitting an appropriate HR increase to help maintain CO in the face of a reduced SV. Collectively, this supports a model in which the remimazolam group exhibited a better effect: a milder initial hemodynamic perturbation was effectively compensated by an HR increase, thereby preventing hypotension. The propofol group, in contrast, exhibited a larger initial insult met with a blunted compensatory response, culminating in more frequent hypotensive episodes.

Our findings provide a novel perspective that helps reconcile the inconsistent literature regarding the hemodynamic profiles of remimazolam and propofol. The existing evidence presents a paradox: while a few studies report superior CO and SVR with remimazolam [32], the majority, including ours and others [28, 33, 34], find comparable steady-state values. Similarly, the reported incidence of hypotension varies considerably across trials [35–37]. We propose that these discrepancies can be resolved by considering three key factors: patient susceptibility, anesthetic technique, and monitoring methodology.

First, patient-specific factors, particularly age and vascular compliance, set the baseline hemodynamic reserve. Studies enrolling younger cohorts may unmask remimazolam’s inherent stability, as their compensatory mechanisms can fully capitalize on its milder perturbation. Conversely, in older patients with intrinsically compromised vascular autoregulation, the modest advantage of remimazolam may be insufficient to overcome a profound age-related decline in reserve, leading to comparable hypotension rates between agents.

Second, the anesthetic regimen itself is a critical determinant. The use of propofol TCI, which achieves a more stable plasma concentration [38], may attenuate the peak hemodynamic insult compared to standard continuous infusion, thereby narrowing the inter-group difference. Furthermore, the synergistic effect of concurrent opioids is pivotal. Protocols employing high-dose remifentanil may potentiate preload reduction, obscuring remimazolam’s benefit and even reversing the SV trend, as seen in Sekiguchi et al. [28]. In contrast, our low-dose remifentanil regimen minimized this synergy, allowing remimazolam’s favorable profile to manifest as a more stable SV and a significant reduction in norepinephrine requirement.

Finally, monitoring methodology dictates what is observed. Studies reliant on intermittent spot-check measurements are inherently blind to the transient, yet clinically significant, hypotensive episodes that characterize propofol’s initial insult. By the time a discrete measurement is taken, corrective vasopressors have often already normalized the pressure, resulting in a false-negative record of stability. Our use of continuous monitoring captured these dynamic fluctuations directly, objectively quantifying the greater initial perturbation with propofol and revealing the protective "hemodynamic buffer" afforded by remimazolam. In summary, the heterogeneity in the literature should not be interpreted as a negation of remimazolam’s utility, but rather as a clarification of the optimal management strategy for hemodynamic stability.

A pertinent finding requiring specific discussion is the observation of higher BIS values in the remimazolam group at several time points. This disparity is unlikely to confound our primary hemodynamic conclusions, given several supporting lines of evidence. First, and most critically, although a statistical difference in BIS was noted, all values in both groups were rigorously maintained within the target anesthesia depth range (40–60), indicating comparable clinical depth. This is further substantiated by the absence of any corresponding significant differences in MAP, CO, or SVR at the time points where BIS differed, creating a clear temporal dissociation between neurophysiological and hemodynamic parameters. Second, the observed BIS pattern is consistent with the known pharmacodynamic profile of remimazolam. Existing literature consistently reports that BIS values under remimazolam are inherently 5–10 points higher than those under propofol at equivalent levels of clinical sedation [15]. Therefore, we interpret the higher BIS values not as an indicator of materially lighter anesthesia, but as a predictable reflection of the distinct neurophysiological profiles of the two agents.

The clinical implications of our findings are substantial. In hypertensive patients, even transient intraoperative hypotension is strongly associated with serious postoperative complications, including myocardial ischemia, acute kidney injury, and stroke [39–41]. A strategy that proactively minimizes these transient episodes, such as using remimazolam for anesthesia induction and maintenance, holds significant potential for improving patient outcomes, particularly in high-risk subgroups. Our findings underscore that achieving superior stability extends beyond the choice of anesthetic agent alone. It all comes down to taking a comprehensive approach. First, choose pharmacologically milder agents like remimazolam, with an inherent "hemodynamic buffer" property, which helps maintain stability to a certain extent. Next, a slow and titrated infusion rather than a bolus may minimize the initial hemodynamic perturbation. Finally, utilizing continuous monitoring capable of guiding real-time interventions and capturing clinically meaningful, albeit transient, fluctuations. This integrated strategy is paramount for vulnerable patients, for whom even statistically non-significant reductions in hemodynamic variability may translate into crucial clinical benefits.

This study has several limitations. First, a limitation inherent to clinical anesthesia research is the difficulty in maintaining a perfectly constant anesthetic depth. While we strictly targeted a BIS of 40–60, brief fluctuations are unavoidable during surgery. We believe this reflects real-world conditions and is unlikely to have systematically biased the comparison between the two groups. Second, while our primary outcome was the hypotension episodes, and supplementary measures such as the amount of norepinephrine usage was reported, our study is limited by the lack of more integrated metrics such as the time-weighted area under the curve for hypotension. Future studies should aim to incorporate these more sensitive and comprehensive endpoints. Third, the use of flumazenil in the remimazolam group was determined by individual anesthesiologists’ clinical judgment rather than a fixed objective criterion. While this reflects clinical reality, it may have introduced variability in emergence time data. Fourth, the collection of secondary hemodynamic-related outcome indicators at discrete timepoints may have led to an underestimation of the true differences between groups. Recording these indicators continuously over a targeted post-induction period (e.g., the first 15 min when hemodynamics are most labile) would more accurately capture dynamic fluctuations, potentially revealing greater disparities in secondary outcomes between propofol and remimazolam. We plan to adopt this methodological adjustment in future research.

In conclusion, utilizing continuous hemodynamic monitoring, this study demonstrates that remimazolam provides superior hemodynamic stability compared to propofol in hypertensive patients undergoing non-cardiac surgery, as evidenced by significantly fewer hypotensive episodes and reduced norepinephrine requirements. Our data suggest a plausible mechanistic model for this clinical advantage: remimazolam appears to cause a smaller initial hemodynamic perturbation and better preserve compensatory HR elevation. These findings highlight the critical importance of assessing dynamic hemodynamic changes, beyond steady-state values, in vulnerable populations. Future research is warranted to determine if these stabilizing effects improve long-term clinical outcomes.

## Data Availability

The datasets generated during the current study are available from the corresponding author on reasonable request.
